# Differential cortical responses to neuromuscular electrical vs. peripheral magnetic stimulation: a multimodal TMS-fNIRS study

**DOI:** 10.3389/fnins.2026.1781058

**Published:** 2026-02-27

**Authors:** Fengyun Yu, Weining Wang, Leyi Xu, Sijie Liang, Ruiping Hu, Yulian Zhu

**Affiliations:** 1 Department of Rehabilitation Medicine, The First Affiliated Hospital of Wenzhou Medical University, Wenzhou, Zhejiang, China; 2 Department of Rehabilitation Medicine, Huashan Hospital, Fudan University, Shanghai, China; 3 Department of Rehabilitation Medicine, Affiliated Hospital of Xuzhou Medical University, Xuzhou, Jiangsu, China

**Keywords:** cortical activity, cortical hemodynamics, functional near-infrared spectroscopy, neuromuscular electrical stimulation, peripheral magnetic stimulation

## Abstract

**Objective:**

To investigate cortical modulatory effects of neuromuscular electrical stimulation (NMES) and peripheral magnetic stimulation (PMS) applied to the wrist extensors of healthy adults, using fNIRS as the primary assessment modality.

**Methods:**

In a randomized crossover design, 15 right-handed adults received NMES and PMS sessions (separated by ≥48 h). Stimulation intensity was functionally calibrated to elicit a matched, maximal painless wrist dorsiflexion. Corticospinal excitability was assessed via motor evoked potentials (MEPs) before and after each intervention. Real-time cortical hemodynamics were monitored with functional near-infrared spectroscopy (fNIRS) during stimulation, quantifying changes in oxygenated ([HbO]) and deoxygenated ([HbR]) hemoglobin concentrations across the sensorimotor (SMC), prefrontal (PFC), and occipital (OC) cortices.

**Results:**

Neither NMES nor PMS induced significant changes in MEP amplitude (NMES: *p* = 0.674; PMS: *p* = 0.794). However, fNIRS revealed fundamentally distinct cortical activation patterns during stimulation. NMES was associated with widespread decreases in [HbO] within the PFC, ipsilateral SMC, and OC (*p* < 0.05). In contrast, PMS elicited focal activation in the contralateral SMC, characterized by a significant increase in [HbO] (ch23: *p* = 0.005; ch35: *p* = 0.022) and a concurrent decrease in [HbR] (*p* < 0.05) compared to the NMES condition. General linear model analysis confirmed more robust contralateral SMC activation during PMS. No significant differences in task-based functional connectivity were observed between the two modalities.

**Conclusions:**

A single session of NMES and PMS differentially modulates real-time cortical hemodynamics without altering corticospinal excitability. PMS induces focal, excitatory-dominant activation of the contralateral SMC, while NMES evokes a pattern of widespread cortical modulation, reflecting their distinct afferent mechanisms.

## Introduction

Neuromuscular electrical stimulation (NMES) is a well-established, non-invasive technique that induces muscle contractions through transcutaneous depolarization of motor axons (Knutson et al., 2025; Olsen et al., 2020; Guo et al., 2018; Bao et al., 2020). Beyond its direct peripheral motor effects, accumulating evidence indicates that NMES also exerts substantial central neuromodulatory influences (Carson and Buick, 2019). Neurophysiological studies employing transcranial magnetic stimulation (TMS) and electroencephalography have demonstrated that NMES delivered to intrinsic hand muscles can modulate excitability within the primary somatosensory (S1) and primary motor (M1) cortices (Espeit et al., 2023; Guo et al., 2018; Insausti-Delgado et al., 2020), processes that are closely associated with cortical reorganization and functional recovery (Barker et al., 2012). Accordingly, NMES is increasingly regarded not merely as an assistive modality for movement execution, but as a potential driver of use-dependent neuroplasticity in neurological rehabilitation (Liu and Au-Yeung, 2017; Young, 2015).

Peripheral magnetic stimulation (PMS) has emerged as a promising alternative to NMES (Chen et al., 2023). By generating pulsed magnetic fields that induce depolarizing currents in deep peripheral tissues, PMS achieves physiological effects comparable to those of NMES while offering distinct practical and mechanistic advantages (Qi et al., 2023; Beaulieu and Schneider, 2013). As a non-contact modality, PMS is typically painless and avoids issues related to skin impedance and electrode placement (Gallasch et al., 2015). More importantly, from a neurophysiological perspective, PMS is hypothesized to elicit a relatively “purer” proprioceptive afferent volley. This arises from both the activation of muscle spindles and Golgi tendon organs through evoked muscle contractions and the direct depolarization of deep sensory nerve fibers by the induced electric field (Gallasch et al., 2015). Such differences in afferent recruitment suggest that NMES and PMS may engage cortical networks through partially distinct pathways, with potentially important implications for their therapeutic mechanisms (Beaulieu et al., 2017; Lampropoulou et al., 2012).

A precise characterization of the immediate cortical effects induced by peripheral stimulation is essential for optimizing intervention strategies. This requires methodological approaches capable of concurrently assessing both corticospinal output excitability and spatially resolved cortical dynamics. TMS remains the gold standard for probing corticospinal pathway integrity, whereas functional near-infrared spectroscopy (fNIRS) provides a complementary, non-invasive means of monitoring task-related cortical hemodynamic activity with high temporal resolution and tolerance to movement (Yang et al., 2019; Wei et al., 2020; Hramov et al., 2020). The combined application of these techniques enables a comprehensive assessment of stimulation-induced neurophysiological responses at both the corticospinal and cortical network levels.

Despite their shared goal of promoting neuroplasticity, direct comparisons of the immediate cortical responses elicited by NMES and PMS remain limited (Keesukphan et al., 2025; Qi et al., 2023). In particular, it remains unclear whether their distinct afferent recruitment profiles translate into differential patterns of cortical activation and corticospinal modulation. Addressing this knowledge gap is critical for refining stimulation-based rehabilitation protocols. Therefore, the present study aimed to directly compare the effects of a single session of NMES and PMS, applied to the wrist extensors under functionally matched intensities, on corticospinal excitability, assessed using TMS, and on real-time cortical hemodynamic activity, measured using fNIRS, in healthy adults. By integrating these complementary neurophysiological measures, this study sought to elucidate the modality-specific central mechanisms underlying two widely used peripheral stimulation techniques.

## Materials and methods

### Participants

Fifteen healthy right-handed volunteers (7 males; mean age 27.13 ± 4.52 years) participated in this exploratory crossover study from August 2020 to January 2021. All participants were free of neurological, psychiatric, or upper limb sensorimotor disorders and had no contraindications to TMS. The study protocol was approved by the Institutional Review Board of Huashan Hospital, Fudan University (reference number: #2019-609), and written informed consent was obtained from all participants prior to enrolment.

### Experiment design

**Figure 1:**
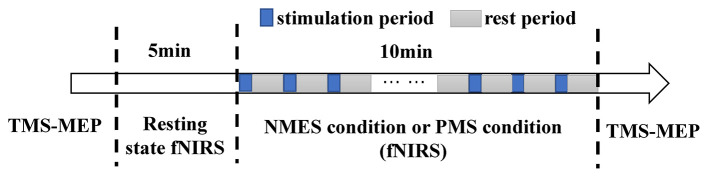
Study timeline. Each session began with baseline TMS-MEP and resting-state fNIRS assessments, followed by the application of either NMES or PMS while fNIRS was recorded, and concluded with a post-intervention TMS evaluation.

The study was conducted in a separate and quiet room. Each participant completed two experimental sessions in a randomized order: one involving NMES and the other PMS applied to the right wrist extensors. Sessions were separated by a washout period of at least 48 h to minimize carryover effects. The experimental timeline is summarized in Figure 1.

At the beginning of each session, a 5-min resting-state fNIRS recording was acquired. Within each session, the following sequence was performed: (1) a pre-intervention assessment of corticospinal excitability using TMS; (2) the peripheral stimulation intervention (NMES or PMS), during which continuous fNIRS data were recorded; and (3) an immediate post-intervention TMS assessment. Participants remained seated comfortably with their eyes open and were instructed to stay relaxed throughout. Subjective comfort was rated after each stimulation session.

### Peripheral muscle stimulation intervention

NMES was delivered using a biphasic constant-current stimulator (ES-521, ITO Co., Ltd, Tokyo, Japan) via a single channel. Stimulating electrodes were placed distal to the common extensor origin and halfway down the extensor surface of the right forearm, covering both extensor carpi ulnar and extensor carpi radialis muscles. The protocol was conducted at a frequency of 50 Hz with 15 repetitions of 10 s on/30 s off stimulation for 10 min, ramp-up and ramp-down taking 1 s. The current intensity was individually adjusted to elicit maximal, visually confirmed wrist dorsiflexion that was comfortable and pain-free (mean± SD: 12.30 ± 3.78 mA).

PMS was applied using a monophasic magnetic stimulator (OSF-pTMS, O.SELF Company, Wuhan, China) with a figure-of-eight coil (outer diameter 70 mm). The coil was held tangentially over the muscle belly of the right forearm extensors to induce maximal wrist dorsiflexion. The stimulation protocol matched the NMES timing structure: 10 Hz pulses delivered in 15 blocks of 10 s on/30 s off, yielding a total of 1,500 pulses over 10 min. Intensity was titrated from a low baseline (e.g., 20% of maximum stimulator output, %MSO) upward to the lowest level that consistently produced a maximal, painless wrist dorsiflexion, with its amplitude visually matched to that achieved during NMES (mean ± SD: 30.47 ± 4.78 %MSO). This titration to the lowest effective intensity mitigates any risk of overstimulation, which is pertinent given the absence of cutaneous discomfort with PMS.

### Transcranial magnetic stimulation assessments

Corticomotor excitability was assessed using single-pulse TMS delivered with an OSF-pTMS magnetic stimulator (O.SELF Company, Wuhan, China) with a figure-of-eight coil. Surface electromyography was recorded from the right first dorsal interosseous (FDI) muscle using Ag/AgCl electrodes placed in a belly–tendon montage. The raw EMG signal was amplified, band-pass filtered (20–1,000 Hz), and sampled at 5,000 Hz for offline analysis. The coil was placed tangentially over the left M1 at a 45° angle to the midline to induce a posterior-anterior current. The hotspot for eliciting Motor evoked potential (MEP) in the FDI was identified by systematically moving the coil in 5 mm increments and was marked on a tight-fitting electrocap to ensure consistent coil placement across pre- and post-intervention measurements.

Resting motor threshold (RMT) was defined as the minimum intensity required to produce MEPs with a peak-to-peak amplitude >50 μV in at least 5 out of 10 consecutive trials in the relaxed FDI muscle. To quantify corticospinal excitability, MEP amplitude was assessed at a fixed suprathreshold stimulus intensity. This intensity was individually determined at baseline as the level required to evoke an approximately 1 mV peak-to-peak MEP amplitude. The same stimulus intensity was then maintained for both pre- and post-intervention assessments to ensure a stable and sensitive measure of changes in corticospinal excitability. At each time point, 10 single-pulse TMS stimuli were delivered with an inter-stimulus interval of at least 5 s.

### Functional near-infrared spectroscopy acquisition and analysis

#### Data acquisition

**Figure 2:**
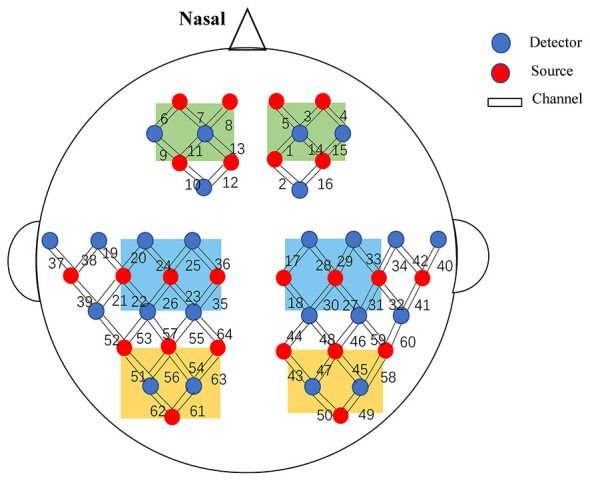
fNIRS optode placement and region-of-interest (ROI) assignment. Optodes were arranged with a source-detector separation of 3 cm. Channels were defined based on MNI coordinates: prefrontal cortex (green), sensorimotor cortex (blue), and occipital cortex (yellow). Key channels over the forearm motor cortex were 23 & 35 **(left)** and 18 & 30 **(right)**. Of the 64 total channels, data from 40 channels (within the colored regions) were used for analysis.

Hemodynamic activity was recorded using a continuous-wave, 64-channel fNIRS system (NirSmart, Danyang Huichuang Medical Equipment, China) with wavelengths of 730 nm and 850 nm at a sampling rate of 11 Hz. The optode array included 24 sources and 24 detectors arranged symmetrically over the prefrontal cortex (PFC), sensorimotor cortex (SMC), and occipital cortex (OC), forming 64 measurement channels with a source–detector separation of 3 cm, corresponding to a cortical penetration depth of approximately 2–3 cm (Figure 2).

### Preprocessing and analysis

fNIRS data were preprocessed and analyzed using HomER2 (version 2.8), a MATLAB-based graphical interface for processing continuous-wave NIRS data (Huppert et al., 2009). The raw optical intensity signals were processed through a standardized pipeline. First, the hmrIntensity2OD function was applied to convert the raw intensity signals into optical density (OD). Motion artifacts were then identified using the HmrMotionArtifactByChannel algorithm with thresholds set at STDev = 10 and AMP = 5, and corrected via spline interpolation. A bandpass filter (0.01–0.1 Hz) was applied to remove low-frequency drift and high-frequency physiological noise. Subsequently, the hmrOD2Conc function was employed to convert OD data into concentration changes of oxygenated ([HbO]) and deoxygenated ([HbR]) based on the modified Beer–Lambert law. For task-based analysis, trial-wise concentration changes of [HbO] and [HbR] were baseline-corrected by subtracting the mean signal from the 5 s interval immediately preceding each stimulation block. Hemodynamic responses were then time-locked to stimulus onset and block-averaged across all trials within each condition using the hmrBlockAvg function, yielding mean response time courses from −5 s to 40 s relative to stimulus onset.

### GLM and network analysis using NirSpark

For subsequent general linear model (GLM) and functional network analyses, data were processed within the NirSpark toolbox (NirSmart, Danyang Huichuang Medical Equipment, China) in the MATLAB environment. Preprocessing in NirSpark applied parameters consistent with the HomER2 pipeline (motion correction: STDev-thresh = 10, AMP-thresh = 5; bandpass filter: 0.01–0.1 Hz; partial pathlength factor = 6). The GLM was implemented to estimate condition-specific [HbO] responses at both individual subject and group levels, yielding beta values for each condition. A 4 s full-width-at-half-maximum Gaussian smoothing kernel was applied to the [HbO] time series to suppress short-duration, high-frequency noise.

Functional connectivity was assessed by constructing brain networks based on inter-nodal correlation. For the 64-channel configuration, Pearson correlation coefficients were computed between all channel pairs. Network edges were defined by applying a series of similarity thresholds (*r* ≥ 0.5, 0.6, 0.7, and 0.8). ROI-based and channel-based connectivity matrices were generated for both NMES and PMS conditions during stimulation. Differences in connectivity strength (ROI-ROI and channel-channel) between the two stimulation conditions were statistically compared using paired *t*-tests, performed separately for the resting-state and task-period data.

### Regions of interest (ROI) definition

Based on standard Montreal Neurological Institute (MNI) spatial coordinates corresponding to the fNIRS source-detector array, channels were grouped into six anatomical ROIs: Left PFC: channels 6, 7, 8, 9, 11, 13; Right PFC: channels 1, 3, 4, 5, 14, 15; Left SMC: channels 20, 22, 23, 24, 25, 26, 35, 36; Right SMC: channels 17, 18, 27, 28, 29, 30, 31, 33; Left OC: channels 51, 54, 56, 61, 62, 63; Right OC: channels 43, 45, 47, 49, 50, 58.

Given the study's focus on cortical responses to peripheral wrist extensor stimulation, channels located over the primary motor representation of the forearm were selected as seeds for detailed analysis. According to MNI coordinates, channels 23 and 25 corresponded to the left forearm motor cortex, and channels 18 and 30 to the right forearm motor cortex.

### Statistical analysis

Statistical analyses were performed using IBM SPSS Statistics (version 22.0). All continuous data are expressed as mean ± standard deviation, with normality confirmed by the Shapiro-Wilk test. Corticospinal excitability was quantified using raw MEP amplitudes and, where appropriate, expressed as percentage change relative to the pre-intervention value (MEP% of baseline) to facilitate within-subject comparisons across stimulation conditions. Additionally, paired *t*-tests were conducted to compare MEP% of baseline values between the NMES and PMS conditions. For fNIRS data, mean concentrations of [HbO] and [HbR] were calculated for the stimulation period (5–10 s post-stimulus onset) and the subsequent rest period (35–40 s post-stimulus onset). Regional activity within each ROI was derived by averaging signals across all constituent channels. Paired *t*-tests were used to compare [HbO] and [HbR] between stimulation and rest periods within each ROI and condition. Direct comparisons between NMES and PMS were performed for stimulation-period [HbO] and [HbR] values within SMC channels. Pearson's correlation analyses were conducted to examine associations between MEP changes and stimulation intensity, between MEP changes and RMT, and between MEP% of baseline values across the two stimulation conditions. To account for multiple comparisons, the Benjamini–Hochberg procedure was applied to control the false discovery rate. Statistical significance was set at a two-tailed *p* value < 0.05.

## Results

All fifteen participants (mean age 27.13 ± 4.52 years) completed the study. Demographic characteristics are summarized in Table 1. No participant reported pain or discomfort during either the NMES or PMS sessions.

**Table 1:** Basic characteristics of subjects in the NMES and PMS conditions.

**Condition**	***N***	**RMT (%MSO)**	**Intervention intensity**	**Pre-MEP (mV)**	**Post-MEP (mV)**	**Paired *t*-test (MEP pre-post)**
NMES	15	37.73 ± 11.81	12.30 ± 3.78 (mA)	1.11 ± 0.32	1.22 ± 0.48	*t* = 0.994; *df* = 14; *p* = 0.674
PMS	15	36.6 ± 12.94	30.37 ± 4.78 (%MSO)	1.12 ± 0.29	1.15 ± 0.51	*t* = 0.266; *df* = 14; *p* = 0.794
Paired *t*-test (NMES-PMS)		*t* = 0.398; *df* = 14; *p* = 0.697		*t* = 0.129; *df* = 14; *p* = 0.900	*t* = 0.574; *df* = 14; *p* = 0.575	

RMT, resting motor threshold; NMES, neuromuscular electrical stimulation; PMS, peripheral magnetic stimulation; MEP, motor evoked potential; MSO%, Maximum stimulator output%.

### Changes in cortical excitability

**Figure 3:**
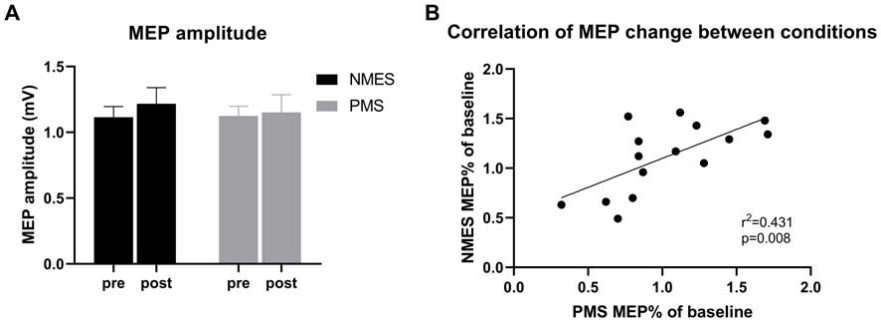
Effects of NMES and PMS on MEPs. **(A)** MEP amplitudes recorded before and after the application of NMES and PMS to the right wrist extensors (mean ± SEM). **(B)** Correlation between the NMES- and PMS-induced changes in MEP amplitude (ΔMEP).

RMT measured prior to stimulation did not differ significantly between the NMES (37.73 ± 11.81 %MSO) and PMS (36.60 ± 12.94 %MSO) sessions (*p* = 0.697). A significant positive correlation was observed between individual RMT measured in the two sessions (*r* = 0.656, *p* = 0.008), indicating good within-subject consistency (Figure 3B).

MEP amplitudes recorded before and after each intervention are presented in Figure 3A. No significant change in MEP amplitude was observed NMES (*p* = 0.674) or PMS (*p* = 0.794). Consistent with these findings, the relative change in MEP amplitude did not differ significantly between the two stimulation conditions.

### fNIRS responses between different stimulation conditions

**Figure 4:**
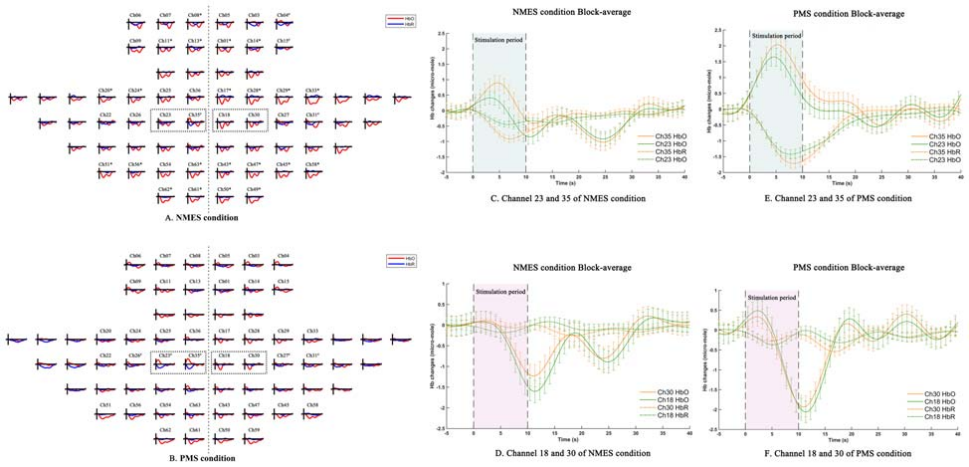
Averaged hemodynamic responses and time courses for NMES and PMS. **(A,B)** Group-averaged (0–40 s) changes in [HbO] (red) and [HbR] (blue). **(A)** NMES elicited significant [HbO] decreases in the PFC, right SMC, and OC (*p < 0.05, Benjamini-Hochberg corrected). **(B)** PMS showed a non-significant trend of [HbO] increase in the left SMC and decrease in contralateral SMC and OC. **(C–F)** Block-averaged time series for specific motor cortex channels under each condition: left channels 23 & 35 during NMES **(C)** and PMS **(D)**; right channels 18 & 30 during NMES **(E)** and PMS **(F)**. The experimental timeline is indicated. Analysis was restricted to channels within the defined ROIs (PFC, SMC, OC). *pHbO < 0.05, ^#^*p*HbR < 0.05.

Resting-state functional connectivity analysis revealed no significant differences in either ROI-ROI or channel-channel connectivity strength between the NMES and PMS conditions prior to stimulation, indicating comparable baseline network organization across sessions. Task-evoked hemodynamic responses are in Figure 4.

During NMES, a significant reduction in [HbO] was observed in the PFC, right SMC, and OC during the stimulation period compared with rest (all *p* < 0.05; Figure 4A). In contrast, channels 23 and 35 within the left motor cortex exhibited a non-significant increase in [HbO]. Concurrently, [HbR] levels was significantly reduced in selected channels within the right PFC (ch4: *p* = 0.020; ch15: *p* = 0.013) and bilateral SMC (left ch35: *p* = 0.010, right ch31: *p* = 0.001).

During PMS, [HbO] in the left SMC demonstrated an increasing trend, whereas decreases were observed in the OC and right SMC. However, none of these [HbO] changes survived correction for multiple comparisons (*p* > 0.05; Figure 4B). In contrast, [HbR] exhibited significant reductions in multiple SMC channels during stimulation relative to rest, indicating a more spatially localized but consistent deoxygenation response under PMS.

**Figure 5:**
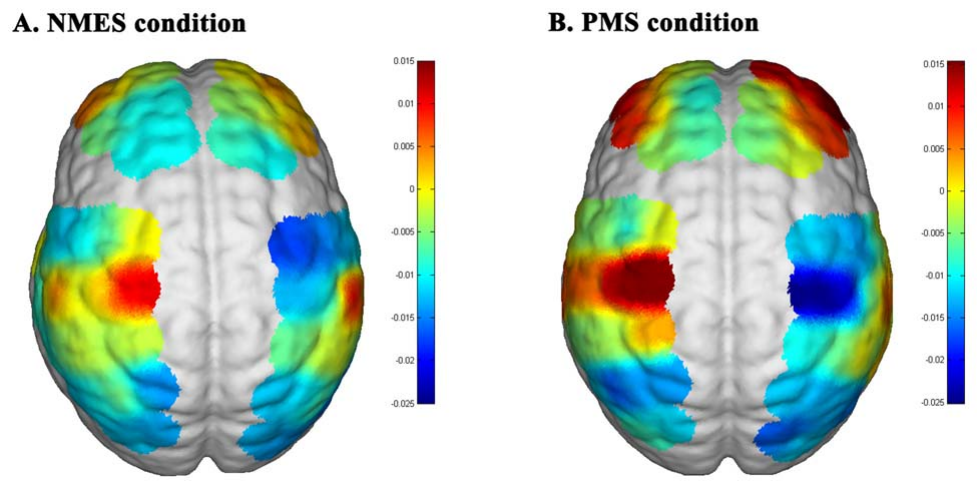
Cortical activation maps. HbO activation (beta scores) maps during **(A)** NMES and **(B)** PMS stimulation. The picture comes from the group GLM analysis of the fNIRS data during stimulation task using Nirspark.

Direct comparison between stimulation modalities revealed distinct hemodynamic patterns within the SMC channels. Specifically, [HbO] concentrations in the left motor cortex (channels 23 and 35) were significantly higher during PMS than during NMES (ch23: *p* = 0.005; ch35: *p* = 0.022). In parallel, [HbR] levels were significantly lower under PMS at several SMC channels (ch23: *p* = 0.007; ch26: *p* = 0.012; ch35: *p* = 0.039), further highlighting modality-specific differences in cortical oxygenation dynamics (Figures 4C–F). GLM-derived [HbO] activation maps are presented in Figure 5. Both NMES and PMS elicited a lateralized activation pattern within the SMC, characterized by positive activation in the left motor cortex accompanied by relative deactivation in the right hemisphere. Notably, the magnitude of [HbO] activation in the left motor cortex was visibly greater during PMS than during NMES, indicating a stronger task-related hemodynamic response under PMS.

### Functional connectivity during stimulation

**Figure 6:**
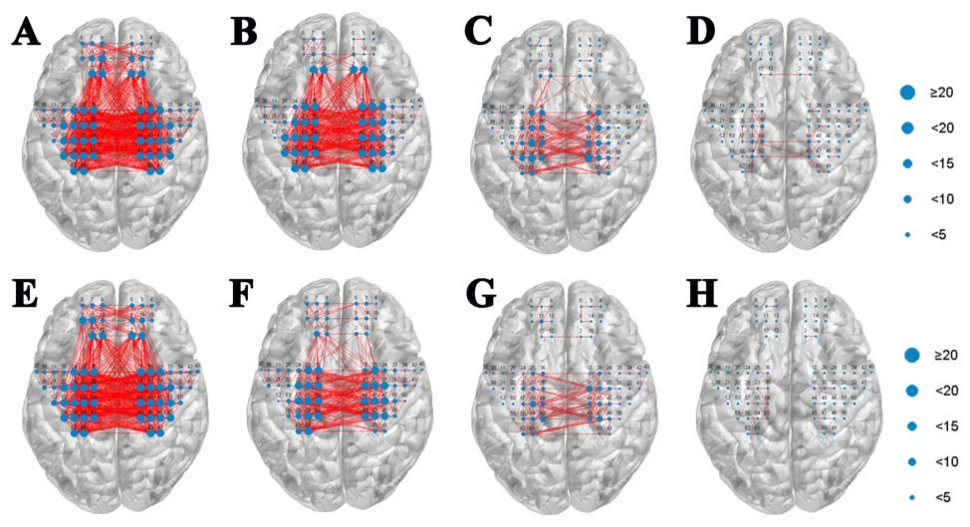
Seed-based correlation analysis. Comparison of the number of functional connection edges between **(A–D)** NMES and **(E–H)** PMS conditions at thresholds of 0.5, 0.6, 0.7, and 0.8.

Task-related functional connectivity analysis revealed no significant differences between the NMES and PMS conditions. Specifically, neither the overall strength of ROI-ROI connections nor the number of significant channel-channel connections differed between stimulation modalities across similarity thresholds ranging from 0.5 to 0.8 (Figure 6).

## Discussion

This multimodal study employed TMS and fNIRS to compare the immediate cortical effects of a single session of NMES and PMS applied to the dominant wrist extensors muscles in healthy adults. Under the specific stimulation parameters employed, no significant after-effects on corticospinal excitability were observed. However, fNIRS revealed markedly distinct task-related hemodynamic response patterns during stimulation: PMS induced focal activation in the contralateral SMC, whereas NMES was associated with widespread decreases in [HbO] in the PFC, ipsilateral SMC, and OC.

To enable a meaningful comparison between these two physiologically distinct stimulation modalities, a key methodological strategy was employed: stimulation intensity was functionally calibrated to elicit a maximal, painless wrist dorsiflexion, rather than matched by device-specific output units. This design was based on two primary rationales. From a physiological perspective, the central modulatory effects induced by peripheral stimulation are primarily driven by the sensory afferent feedback associated with motor output, rather than the absolute physical intensity of the stimulus (Pohjonen et al., 2022). Calibrating intensity to achieve comparable motor output therefore aimed to ensure a higher degree of equivalence in the biological signals relayed to the central nervous system. From a methodological standpoint, this strategy minimized potential confounding effects arising from differential pain perception, attentional allocation, or inconsistent movement amplitude (Rossini et al., 2015). Although the absolute physical intensities of the two modalities are not directly comparable, this approach achieved a high level of consistency in participant experience and peripheral motor activation. Consequently, the observed differences in cortical activation patterns are more likely to reflect fundamental disparities in the recruitment properties of afferent nerve fibers inherent to each stimulation modality, rather than differences in peripheral activation strength or subjective sensation.

### Absence of after-effects on cortical excitability

No significant changes in MEP amplitude were observed following either a single session of NMES or PMS. This finding should be interpreted within the context of the specific stimulation parameters employed. Previous studies have established a clear dose–response relationship for NMES-induced cortical excitability changes. Sasaki et al. (2017) reported that 20 min of median nerve NMES at 30 Hz significantly increased MEP amplitude when delivered above MT, whereas stimulation at 90% of MT was ineffective. Similarly, Huang et al. (2019) demonstrated that a significant increase in cortical [HbO], measured by fNIRS, occurred only when NMES current intensity exceeded 20 mA, showing a clear dose-dependent pattern. Furthermore, stimulation duration is critical, as NMES protocols capable of inducing sustained facilitatory effects typically require intervention durations exceeding 20 min (Andrews et al., 2013; Wu et al., 2005). In comparison, the intensity and duration of NMES used in the present study were relatively conservative and may have been insufficient to elicit detectable after-effects.

Similarly, the effects of PMS on cortical excitability are highly parameter-dependent (Gallasch et al., 2015; Nito et al., 2021). Evidence indicates that rPMS delivered at frequencies ≥25 Hz for at least 15 min is effective in enhancing M1 excitability and improving motor performance, whereas 10 Hz rPMS failed to induce changes in cortical excitability (Nito et al., 2021). Moreover, 25 Hz rPMS has been shown to be superior to 10 Hz in inducing long-term potentiation–like plasticity within the SMC (Gallasch et al., 2015) and demonstrates greater clinical efficacy in improving upper limb motor function and reducing hand edema post-stroke (Jiang et al., 2022; Jia et al., 2021; Fujimura et al., 2025). In contrast, inducing measurable after-effects with 10 Hz PMS typically requires significantly prolonged intervention durations, with studies suggesting the need for continuous stimulation of at least 45 min (McKay et al., 2002) or cumulative application times exceeding 2 h (Ridding et al., 2000; Kaelin-Lang et al., 2002). Therefore, the short-duration 10 Hz PMS protocol used in the present study likely did not reach an effective stimulation dose.

Although stimulation was applied to the wrist extensors, MEPs were recorded from the FDI muscle to assess excitability changes across the broader corticospinal system rather than localized effects restricted to the target muscle. Previous studies indicate that peripheral stimulation can induce heterotopic modulation of corticospinal excitability via intracortical network mechanisms within M1 (Yamamoto et al., 2022). While this strategy is suitable for capturing system-level modulation, it may reduce sensitivity to highly focal effects. Thus, the absence of MEP changes in the FDI does not preclude the possibility of more localized excitability changes within the cortical representation of the stimulated muscle.

### Neural mechanisms underlying differential activation patterns induced by NMES and PMS

Despite the absence of lasting excitability changes, the real-time hemodynamic responses captured by fNIRS suggest that the two stimulation modalities engage sensorimotor networks in distinct, modality-specific ways during the intervention.

Under matched peripheral motor output, PMS primarily elicited focal activation in the contralateral SMC, characterized by increased [HbO] and decreased [HbR], representing a typical pattern of enhanced local neurovascular coupling. This focused activation pattern aligns with the physical properties of PMS and its selective recruitment mechanism for deep proprioceptive afferents. Unlike NMES, the time-varying magnetic field generated by PMS penetrates high-resistance skin tissue, preferentially activating deep Ia-class proprioceptive afferent fibers within the muscle while relatively sparing cutaneous receptors (Beaulieu et al., 2017; Lampropoulou et al., 2012). This relatively “pure” proprioceptive input (Beaulieu et al., 2017) is transmitted primarily via the dorsal column and spinothalamic tracts to the contralateral SMC (Struppler et al., 2004; Okudera et al., 2015; Struppler et al., 2007) and can rapidly modulate excitability in this region within approximately 1 s (Sato et al., 2016). The focal activation pattern observed in the present study may therefore reflect the rapid and efficient recruitment of sensorimotor circuits by PMS.

In contrast, NMES elicited only limited activation in the contralateral SMC, accompanied by more widespread decreases in [HbO] across the PFC, ipsilateral SMC, and occipital regions. This hemodynamic pattern should not be simplistically interpreted as cortical deactivation but rather likely reflects the non-selective nature of transcutaneous electrical stimulation. NMES concurrently activates motor axons, low-threshold tactile fibers (Aβ), and smaller-diameter afferent fibers (Aδ and C; Szecsi et al., 2010), resulting in a complex afferent volley integrating proprioceptive, tactile, and potentially nociceptive signals. Within this context, the widespread recruitment of cutaneous afferents may introduce a degree of “non-physiological noise” that competes with task-relevant proprioceptive input derived from muscle contraction, thereby attenuating effective drive to the sensorimotor cortex (Beaulieu et al., 2017). Furthermore, mild nociceptive input may further suppress M1 excitability by engaging pain-modulatory circuits (Carson and Buick, 2019; Tashiro et al., 2019). These mechanisms may collectively account for the relatively constrained activation observed in the contralateral SMC during NMES in this study.

The signal changes observed in the PFC likely reflect the additional cognitive and integrative load required to process this multimodal sensory information (Muthalib et al., 2015). NMES concurrently engages multiple regions including S1, M1, premotor areas, and the PFC (Carson and Buick, 2019). Therefore, the net effect of NMES on cortical activity likely represents a dynamic balance between excitatory proprioceptive drive and inhibitory influences stemming from cutaneous and nociceptive inputs (Struppler et al., 2007; Beaulieu et al., 2017). an interpretation consistent with prior network-level findings. The widespread [HbO] decreases observed herein may thus reflect the hemodynamic signature of large-scale cortical network reconfiguration or inhibitory regulation in response to processing NMES's complex, mixed afferent input.

In summary, the different hemodynamic patterns induced by NMES and PMS in this study are more likely to originate from fundamental differences in the composition of the afferent volley and the characteristics of network recruitment, rather than from differences in stimulation intensity per se.

### Absence of functional connectivity differences

No significant differences in task-based functional connectivity were found between the NMES and PMS conditions. This null finding may be attributed to the relatively short stimulation duration, limited sample size, and the sensitivity constraints of fNIRS-based connectivity analysis in healthy populations. More subtle or distributed network changes might require longer stimulation periods or repeated interventions to become detectable.

Notably, the dissociation between TMS and fNIRS findings underscores the complementary value of a multimodal assessment approach. While TMS-evoked MEPs are considered the gold standard for assessing net corticospinal output excitability, they may lack sensitivity to transient, intra-intervention dynamic cortical processing. In contrast, fNIRS captures real-time hemodynamic changes occurring during stimulation (Huang et al., 2019). The present results suggest that the modality-specific cortical engagement patterns characteristic of each peripheral stimulation technique may precede or occur independently of detectable changes in corticospinal excitability.

### Limitations

This study has several limitations. First, the modest sample size, inherent to its pilot and exploratory nature, limits the statistical power to detect small-to-medium effect sizes and increases the risk of Type II errors. Therefore, caution is warranted when interpreting the negative findings. Second, the single-session design precludes conclusions regarding cumulative or long-term neuromodulatory effects, which are more relevant to clinical rehabilitation applications. Third, fNIRS channel localization was based on standardized anatomical coordinates rather than individual MRI data, which may compromise spatial precision. Finally, the limited penetration depth of fNIRS restricts the assessment of deeper cortical and subcortical structures that may be involved in peripheral stimulation-induced neuromodulation.

### Conclusion

In summary, a single 10-min session of low-intensity NMES or 10 Hz PMS did not produce measurable after-effects on corticospinal excitability under the specific parameters used. However, the two interventions elicited distinct task-related cortical hemodynamic response patterns: PMS induced focal activation of the contralateral SMC, whereas NMES involved a more widespread cortical network encompassing sensorimotor and prefrontal regions.

## Data Availability

The original contributions presented in the study are included in the article/supplementary material, further inquiries can be directed to the corresponding author.
